# Integration of Transcriptomics and Metabolomics Reveals the Antitumor Mechanism Underlying Shikonin in Colon Cancer

**DOI:** 10.3389/fphar.2020.544647

**Published:** 2020-10-22

**Authors:** Yang Chen, Yun Gao, Xiaojiao Yi, Jinghui Zhang, Zhongjian Chen, Yongjiang Wu

**Affiliations:** ^1^ College of Pharmaceutical Sciences, Zhejiang University, Hangzhou, China; ^2^ Institute of Cancer and Basic Medicine (ICBM), Chinese Academy of Sciences, Hangzhou, China; ^3^ Cancer Hospital of the University of Chinese Academy of Sciences, Hangzhou, China; ^4^ Zhejiang Cancer Hospital, Hangzhou, China

**Keywords:** transcriptomics, metabolomics, shikonin, colon cancer, purine metabolism

## Abstract

Colorectal cancer is a common malignancy occurring in the digestive system, which is the third common cause of cancer mortality in developed countries. Shikonin, a naphthoquinone compound extracted from the root of *Lithospermum erythrorhizon*, is extensively reported to exert antitumor activity against various types of cancer. However, the systematic effect of shikonin in colon cancer remains poorly understood. In the present study, we evaluated the antitumor activity of shikonin in human colon cancer cells and the therapeutic effect on a xenograft mouse model. Transcriptomics and metabolomics were further integrated to provide a systematic perspective of the shikonin-induced antitumor mechanism. The results demonstrated that shikonin had a remarkable antitumor potency both *in vitro* and *in vivo*. Moreover, metabolic pathways, including the purine metabolism, amino acid metabolism, and glycerophospholipid metabolism, were perturbed and subsequently led to cell cycle arrest in the G2/M phase. In particular, the disturbance of purine metabolism may account for the major mechanism resulting from shikonin antitumor activity.

## Introduction

Colorectal cancer (CRC) is a common malignancy occurring in the digestive system, and it is reported as the fourth major cause for cancer-related mortality in the world (Gansler et al., 2010). Currently, surgical resection combined with chemotherapy and radiotherapy is still served as the predominant treatment for early- and mid-stage patients with localized colon cancer. However, their side effects are considerable, and the poor prognosis is valued to be addressed (Schlick et al., 2019). Hence, the development of natural products has become a priority for the therapy of colorectal cancer.

Shikonin (SHK), a naphthoquinone pigment isolated from the root of *Lithospermum erythrorhizon* (Sieb. et Zucc, Boraginaceae), has been known as an antitumor natural product varying with tumor types during the past decades (Tan et al., 2011). A diversity of antitumor mechanisms involved in shikonin has been reported, including inhibiting cell proliferation (Huang and Hu, 2018), inducing apoptosis (Zhai et al., 2017), and activating necroptosis (Shahsavari et al., 2018). Considering the excellent antitumor activity of shikonin, it is imperative to discover its molecular pharmacological mechanisms. However, the systematic effect of shikonin in cancer therapy remains poorly understood.

In recent years, omics techniques with advanced analysis tools have been widely utilized in medical diagnostics and basic research, including characterizing complex biosystems and illuminating the therapeutic mechanisms in various diseases (dos Santos et al., 2016). Transcriptomics is mainly performed to identify aberrant gene expression which could provide a deep understanding of the biological system at the gene level (Sager et al., 2015), while metabolomics is extensively applied to comprehensively evaluate dynamic changes of global endogenous metabolites and their perturbed pathways (Dettmer et al., 2007). Through the integration of transcriptomic and metabolomic data sets, the molecular mechanism of drugs and potential metabolic biomarkers for cancers in precision medicine can be better interpreted (Karczewski and Snyder, 2018).

In the current study, we investigated the antitumor activity of shikonin in colon cancer both *in vitro* and *in vivo*. The effects of shikonin on the cell cycle and tubulin interaction in SW620 cells were examined, and profiles of transcriptomic and metabolomic in SW620 colon cancer cells, as well as tumor tissue, were integrated to determine the related biochemical pathways of shikonin. Compared with the previous study, our study elucidated the novel molecular target and provided a more comprehensive perspective for exploring the possible signatures.

## Materials and Methods

### Chemicals and Reagents

Shikonin (purity above 98%) was purchased from Aladdin Biotechnology Co., Ltd. (Shanghai, China) and was dissolved in dimethyl sulfoxide (DMSO). The final concentration of DMSO was controlled not to exceed 0.02% when diluted to the required concentration. Formic acid (FA) was purchased from Sigma-Aldrich (MO, United States). HPLC-grade methanol (MeOH) and acetonitrile (ACN) were obtained from VWR Chemicals (Paris, France). All the reagents and chemicals utilized in this study were of analytical grade. Human colon adenocarcinoma cell lines HT29, HCT116, SW480, and SW620 were purchased from KeyGen Biotech (Nanjing, China). The DAPI and Cell Counting Kit were purchased from Yeasen Biotechnology Co., Ltd (Shanghai, China). The β-tubulin antibody and CoraLite488-conjugated anti-mouse IgG were obtained from Proteintech (Rosemont, United States).

### Cell Culture

The human colon cancer cell lines HT29, HCT116, SW480, and SW620 were cultured in RPMI-1640 (Gibco, United States) containing 10% FBS (Gibco, United States) and 1% penicillin–streptomycin (Gibco, United States) at 37 C in a Lab-Line CO_2_ Incubator (Thermo, United States). And a humidified atmosphere of 95% air and 5% CO_2_ were also controlled.

### Cell Viability Assay

The four cell lines were seeded into 10-cm^2^ dishes (Costar, United States) at a density of 1.0 × 10^6^ cells/ml and cultured at 37 °C for 24 h. After being treated with various concentrations of shikonin (0–5 μM) for 48 h, the Cell Counting Kit-8 (CCK-8) assay was used to assess the proliferation of cells. 10 μl of CCK-8 solution was added to each well of the plate and then incubated for 3 h. The absorbance at 450 nm was finally measured using a microplate reader. Besides, SW620 cells were further incubated with shikonin (0, 0.5, 1.0, and 1.5 μM) for 24, 48, and 72 h to illustrate the cytotoxic effect with different incubated time.

### Cell Cycle Analysis

To determine whether shikonin had an effect on the cell cycle, SW620 cells were seeded in a 6-well plate (Costar, United States) at a density of 1.0 × 10^5^ cells/ml and cultured at 37 °C for 24 h. After being treated with 0, 0.5, and 1.0 μM shikonin for 48 h, cells were collected and fixed in 70% ethanol at –30 °C overnight. Ethanol was discarded, and the cells were washed with PBS. Samples were incubated with DNA staining solution and permeabilization solution according to the Cell Cycle Staining Kit (LIANKE Biotech, Nanjing, Jiangsu). For each sample, 1 × 10^4^ cells were analyzed by using a flow cytometer.

### Immunofluorescence Analysis

SW620 cells were seeded in 6-well chamber slides at a density of 1.0 × 10^5^ cells/ml and cultured overnight. After being treated with vehicle (DMSO), 1.0 μM shikonin, vincristine, and paclitaxel for 48 h, cells were fixed with cold methanol at −20 °C for 10 min, and washed with PBS and 0.1% Triton X-100 for 30 min at 37 °C. Cells were then blocked with 1% BSA for 1 h, washed with PBS for 30 min, and incubated with anti-β-tubulin antibody for 1 h at 37 °C. After being washed with PBS for 30 min, cells were further incubated with CoraLite488-conjugated anti-Mouse IgG for 1 h at 37 °C. Finally, DAPI staining was performed, and images were captured by using a fluorescence microscope (Olympus, Japan).

### Metabolomics Analysis of Cell Extracts

After treatment with different concentrations of shikonin (0, 0.5, 1.0, and 1.5 μM) for 48 h, the SW620 cells were quickly separated from the culture medium, thawed on ice, and 300 μL cold acetonitrile (ACN)/H_2_O (75:25, v/v) was added for intracellular metabolites extraction and then vortexed for 30 sec. The process containing freeze, thaw, and vortex was repeated for three times. After being centrifuged for 15 min, the supernatants were transferred to the new 1.2-ml polypropylene tubes and then kept in a –80 °C refrigerator for 15 min. The frozen samples were dried in a freeze dryer immediately for 3 h. The residues were redissolved in 80 μl ACN/H_2_O (20:80, v/v) and centrifuged for 15 min (13,000 rpm, 4 °C). An aliquot of 60 μl supernatant of each sample was then transferred to a new vial for UHPLC-MS analysis in a random order.

Meanwhile, 15 μl of each redissolved sample was pooled in order to prepare seven quality control (QC) samples. Each QC sample was analyzed periodically in the analytical run sequence to monitor instrument stability.

Moreover, an UltiMate 3000 UHPLC system (Dionex, United States) coupled with a Q Exactive^™^ Hybrid Quadrupole-Orbitrap Mass Spectrometer (Thermo Scientific, Germany) was operated for metabolomic analysis. An ACQUITY UPLC HSS T3 column (2.1 mm × 100 mm × 1.8 μm, Waters, United State) was utilized to separate the metabolites at 40 °C. The mobile phase was composed of solvent A (0.1% formic acid in water) and solvent B (acetonitrile). The flow rate was remained at 0.3 ml/min. The autosampler temperature was set at 4 °C. Gradient elution was adjusted as follows: 0–1 min: 2% B, 1–19 min: 2–100% B, 19–21 min: 100% B, and 21–25 min: 2% B. The injection volume of each sample for analysis is 5 μl. Both ESI (+) and ESI (−) mode were performed by mass spectrometry. And parameters used in current experiment were according to the methodology described by Yi et al. (2018).

### Metabolomics Data Processing and Metabolite Biomarker Identification

Raw data from UPLC-MS were converted into mzXML format utilizing MSConvert tool (http://proteowizard.sourceforge.net/downloads.shhtml) for further analysis. Nonlinear retention time correction, peak filtration, and extraction were performed through the XCMS package of R (v3.4.1). Subsequently, the profile containing mass-to-charge ratio (*m*/*z*), retention time, and ion intensity was further processed by metaX package of R (v3.4.1), in which the signal correction and peak normalization were performed according to the QC samples. Metabolites with coefficient of variation (CV) value >30% in QC samples were excluded for the metabolite discovery. Batch normalization of the peak area was proceeded to compare the data from different samples, and multivariate statistical analysis was performed by SIMCA-P 14.1 software (Umetrics, Sweden) using unit variance scaling and mean-centered methods. Principal component analysis (PCA) and orthogonal partial least square discriminant analysis (OPLS-DA) were applied to discriminate control and drug-treated groups. Metabolites which changed significantly among different groups were screened with variable importance in the projection (VIP) exceeding 1, and FDR < 0.05 were finally identified as differential metabolites. Both METLIN (http://metlin.scripps.edu) and HMDB (http://www.hmdb.ca/) were applied for the identification of filtered metabolites. And part of differential metabolites was further confirmed by matching both MS/MS spectra and the retention time with commercially available standards. Heatmap package in R (v3.4.1) was further used to cluster differential metabolites among all groups. Meanwhile, the relevant metabolic pathways were enriched by MetaboAnalyst 4.0 (http://www.metaboanalyst.ca) as well to discover the significant pathways altered by shikonin.

### Transcriptomics Analysis of Cell Extracts

Transcriptomics was used to evaluate mRNA levels in SW620 cells after treatment with 1.0 μM shikonin for 48 h. Total RNA extraction was performed with the QIAGEN RNeasy kit (Qiagen, United Kingdom) according to the manufacturer’s procedure. One percentage agarose gel was used for monitoring RNA degradation, and the NanoPhotometer spectrophotometer (IMPLEN, CA, United States) was applied for checking contamination. A Qubit^®^ RNA Assay Kit in a Qubit^®^2.0 Fluorometer (Life Technologies, CA, United States) was used to measure the RNA concentration, and an RNA Nano 6000 Assay Kit of the Bioanalyzer 2100 system (Agilent Technologies, CA, United States) was performed to assess the integrity. A total of 3 μg RNA per sample was utilized as input material for the RNA sample preparations. An NEBNext^®^ Ultra^™^ RNA Library Prep Kit for Illumina^®^ (NEB, United States) was used according to the manufacturer’s instructions to generate sequencing libraries. Besides, index codes were added to attribute sequences to each sample. Finally, an AMPure XP system (Beckman Coulter, Beverly, United States) and Bioanalyzer 210 were performed to purify the library fragments preferentially 250–300 bp in length and assess separately. Illumina sequencing by synthesis technology was applied to perform the sequence of the resultant double-stranded cDNA libraries.

After downloading the reference genome and gene model annotation files from genome website in advance, the RNA sequence data analysis was performed by HISAT2 v2.0. Then the mapped reads of each sample were assembled by StringTie (v1.3.3b) in a reference-based approach, and a gene expression level was quantified by Feature Counts v1.5.0-p3. The *p*-values of genes were adjusted by the Benjamini and Hochberg method, and corrected *p*-value (0.05) and absolute fold change (2) were chosen as the threshold for differential expressed genes. KEGG pathway enrichment was performed by R package.

### Integrated Analysis of Metabolomics and Transcriptomics Data

Finally, the data sets of metabolomics and transcriptomics were integrated by MetScape software to examine the potential relationship between differently expressed genes and significantly changed metabolites.

### Animals and Sample Collection

To evaluate the potential effect of shikonin on tumor growth *in vivo*, a xenograft model was established in nude mice using the SW620 cell line. Female nude mice, 7 weeks old, were purchased from the Hangsi Biotechnology Co., Ltd. (Hangzhou, China). The mice were housed and fed following the recommendations of the ethics committee. The nude mice were xenografted with 3 × 10^6^ SW620 colon cancer cells resuspended in 100 μl of PBS and inoculated subcutaneously in the right flank from the back of mice. The experimental procedures were approved by the Zhejiang Cancer Hospital Ethics Committee [license number: SCXK (SU) 2017-0005]. Animals were randomly divided to three groups (*n* = 8), and each group was treated with a single dose of 1 or 2 mg kg^−1^ shikonin (dissolved in 0.5% CMC-Na) and vehicle via intraperitoneal injection once every 2 days for 15 days until the tumors reached a mean of 3 mm × 3 mm. At the end of the experiment, the blood samples from each mouse were collected via the retro-orbital venous plexus, and serum samples were separated and stored at −20°C for biochemical analysis. The tumor masses were dissected and weighed, then rapidly quenched in liquid nitrogen, and stored at −80°C immediately for further analysis.

### Biochemical Analysis

The serum was divided into several aliquots to avoid freezing and thawing. Serum levels of alanine aminotransferase (ALT), aspartate aminotransferase (AST), serum creatinine (Scr), and blood urea nitrogen (BUN) were measured according to the manufacturer’s prescripts using ELISA kits (Nanjing Senberga Biotechnology Co., Ltd., Nanjing, China).

### Metabolomics Analysis of Tumor Tissue

An aliquot of 20 mg of tumor tissues was weighted, and 400 μl cold methanol was added for metabolite extraction. After being ground with breads for 3 min and then centrifuged for 15 min (13,000, 4 °C), the supernatants were subsequently transferred to the new 1.2-ml polypropylene tubes. Then 400 μl cold water was added, and the mixture was subsequently frozen in a –80 °C refrigerator for 15 min. The frozen samples were dried in the freeze dryer immediately for 3 h. The residues were redissolved in 80 μl ACN/H2O (20:80, v/v) and centrifuged for 15 min (13,000, 4 °C). An aliquot of 60 μl supernatant per sample was then transferred to a new vial for UPLC-MS analysis in a random order. The following operation and the condition of instruments corresponded to the cellular metabolomics, except for the gradient elution, which was adjusted as follows: 0–1 min: 2% B, 1–12 min: 2–100% B, 12–15 min: 100% B, and 15–16 min: 2% B.

### Real-Time Quantitative Polymerase Chain Reaction

Total RNA was extracted from culture cells by TRIzol reagent, and PrimeScriptTM II 1st Strand cDNA Synthesis Kit (TaKaRa, Japan) was performed to synthesize cDNA with mRNA-specific primers. The primers for differentially expressed genes and control GAPDH were obtained from Proteintech (Rosemont, United States). Applied Biosystems 7500 Real-Time PCR machine was used for the real-time quantitative PCR (RT-qPCR). The 2^−ΔΔCT^ method was used to determine the relative expression of targeted genes, and all reactions were repeated three times. The sequences of primers used are listed in Supplementary Table S2.

### Statistical Analysis

SPSS 24.0 for Mac (SPSS, Inc.) was used for data analysis. Results were represented as mean ± SD and evaluated by using the two-tailed unpaired Student’s *t*-test or one-way analysis of variance. The *p-*value < 0.05 was considered to be significant.

## Results

### Cytotoxic Effect of Shikonin on Human Colon Cancer Cell Lines

**FIGURE 1:**
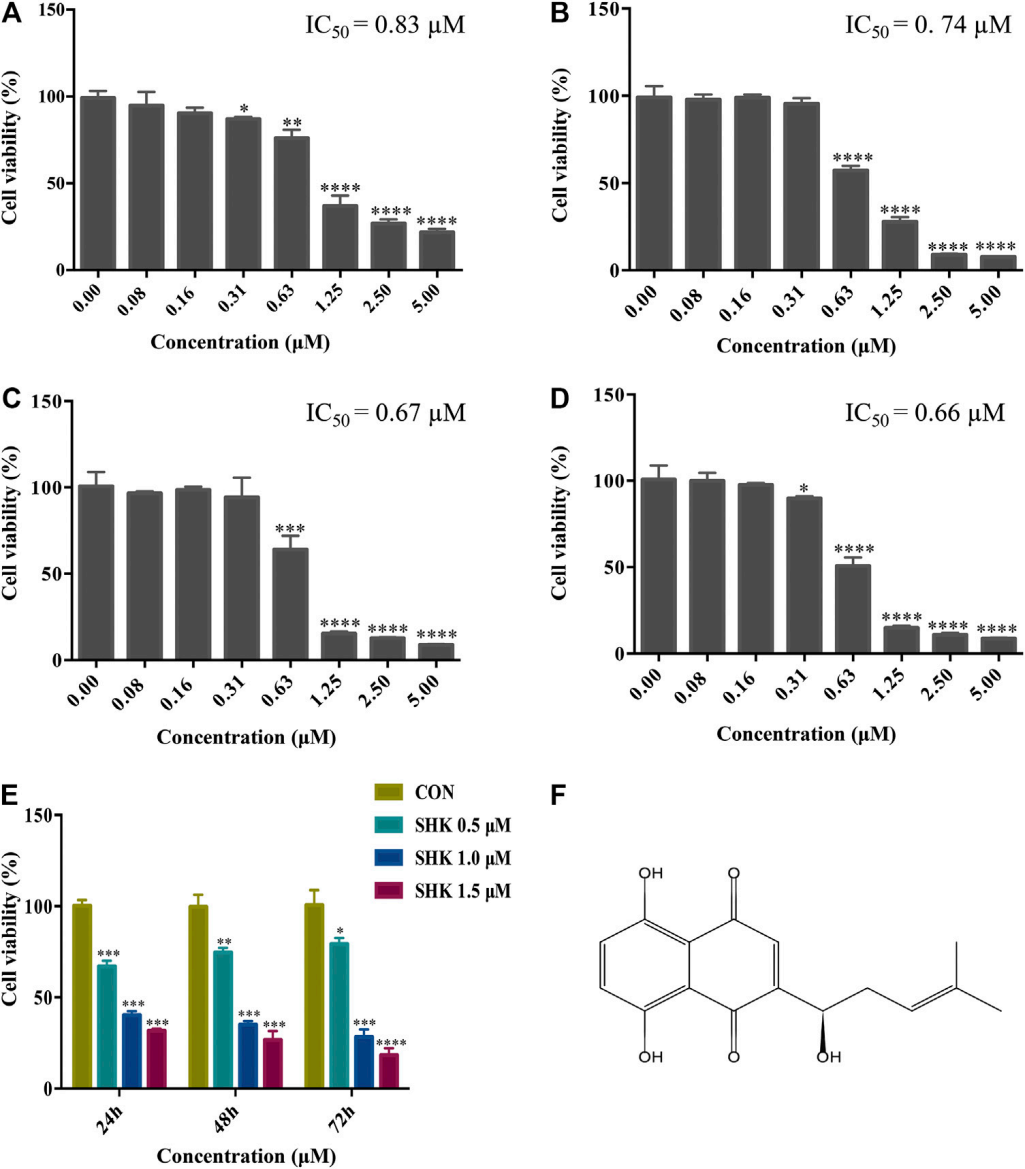
Cytotoxic effect of shikonin on human colon cancer cell lines. **(A)** HT29 cells. **(B)** HCT116 cells. **(C)** SW620 cells. **(D)** SW480 cells. **(E)** Cytotoxic effect of shikonin under the selected dosages on SW620 cells for 24, 48, and 72 h. Data are expressed as mean ± SD (*n* = 3). **(F)** Structure of shikonin.

The cytotoxicity of different concentrations of shikonin was exhibited in four human colon cancer cell lines HT29, HCT116, SW620, and SW480 (Figures 1A–D), which suggested that the significant cytotoxicity exerted by shikonin was in a dose-dependent manner. Meanwhile, SW620 cells treated with the selected concentrations of shikonin for 24, 48, and 72 h revealed that the cell viability of SW620 decreased in a time-dependent manner (Figure 1E). Based on the cell viability results, 1.0 μM for shikonin resulting in 65–70% cell viability was selected for the following transcriptomics and metabolomics study. The structure of shikonin is presented in Figure 1F.

### Effect of Shikonin on SW620 Colon Cancer Cell Cycle Progression

**FIGURE 2:**
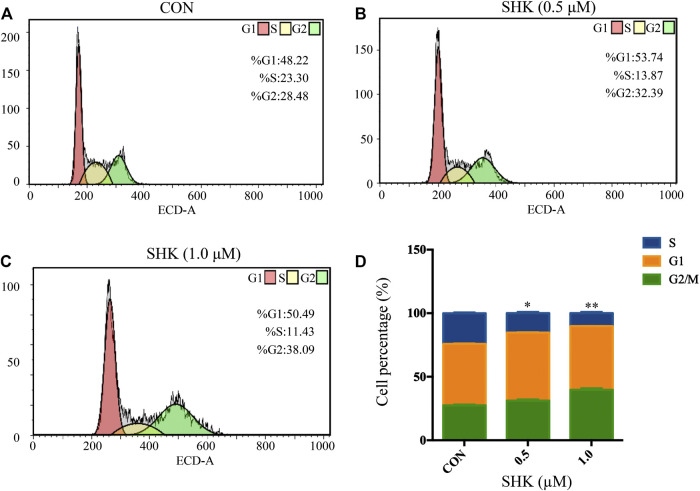
Shikonin-induced cell cycle arrest in SW620 cells. **(A–C)** SW620 cells were treated with shikonin at 0, 0.5, and 1.0 μM for 48 h. Propidium iodide staining and flow cytometry analysis were used to illustrate the distribution of each phase. **(D)** Representative percentage of each cell cycle phase for SW620 cells after exposure to shikonin. Data are expressed as mean ± SD, *n* = 3 (*p < 0.05 and **p < 0.01), compared with control group.

Shikonin contributed to the accumulation of SW620 cells in the G2/M phase in a dose-dependent manner (Figures 2A–C). The bar graphs provided an overview of the percentage of SW620 cells upon shikonin treatment (Figure 2D), and cells in the G2/M phase increased from 28.48% (control) to 32.39 and 38.09% under treatment with shikonin 0.5 and 1.0 μM, respectively.

### Effects of Shikonin on Microtubule Organization

**FIGURE 3:**
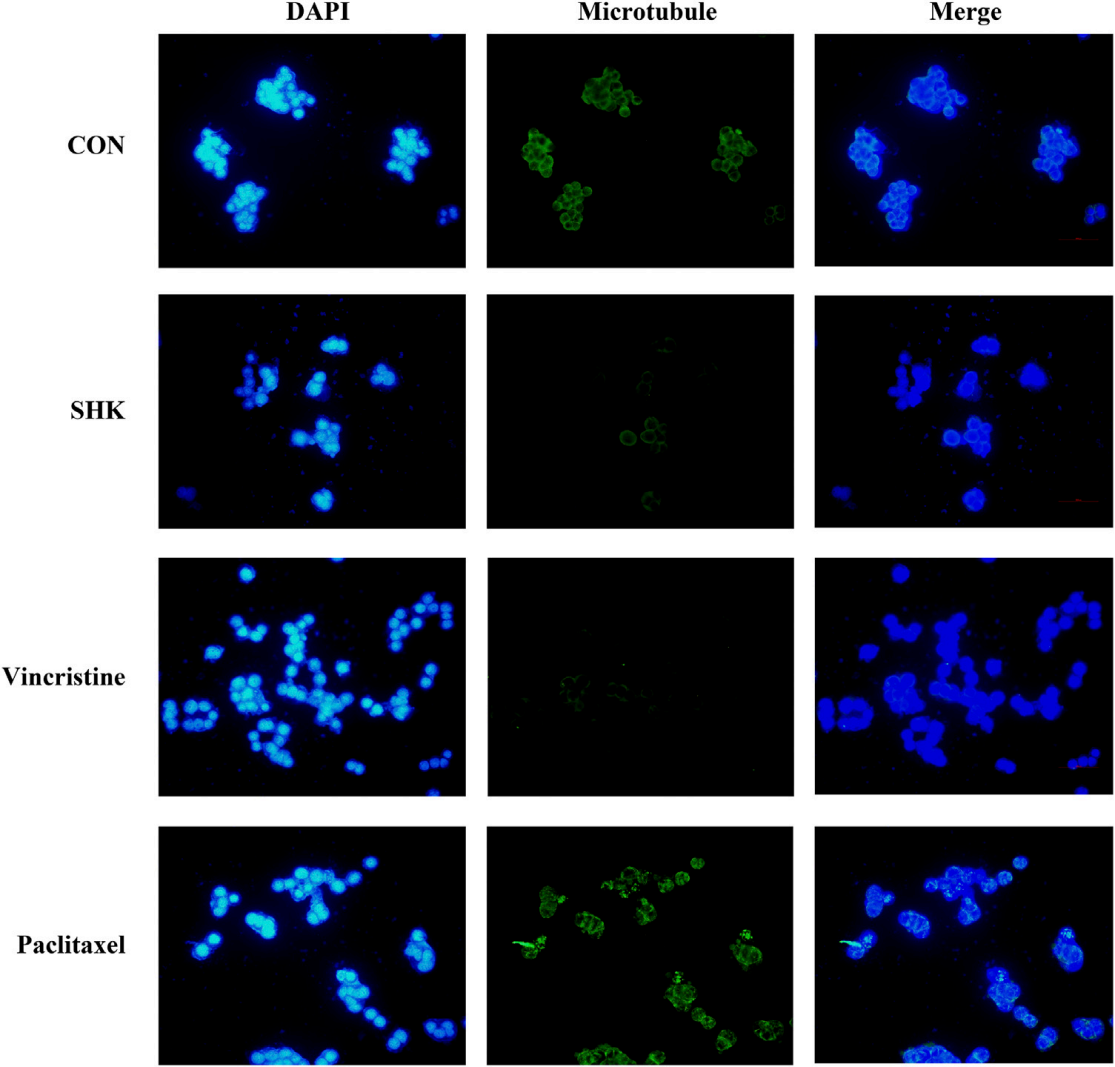
Effect of shikonin on microtubule cytoskeleton in SW620 colon cancer cells. SW620 cells were treated with vehicle (DMSO), 1.0 μM shikonin, paclitaxel, and vincristine for 48 h. Microtubules were stained with β-tubulin antibody and CoraLite488-conjugated anti-mouse IgG. Nuclear DNA was visualized by DAPI staining.

The significant mitotic arrest involved in the G2/M phase was further evaluated by immunofluorescence analysis. Paclitaxel and vincristine, two conventional agents, both targeted on the microtubule for tumor treatment. As shown in Figure 3, the microtubule network arrangement and distribution were disturbed under shikonin treatment in comparison with the control group. Similar to vincristine, a microtubule-destabilizing agent, the disruption of the microtubule cytoskeleton was exhibited by shikonin, and paclitaxel resulted in G2/M cell cycle arrest through stabilizing microtubules.

### Metabolomic Analysis Results of Shikonin-Treated SW620 Colon Cancer Cell

**FIGURE 4:**
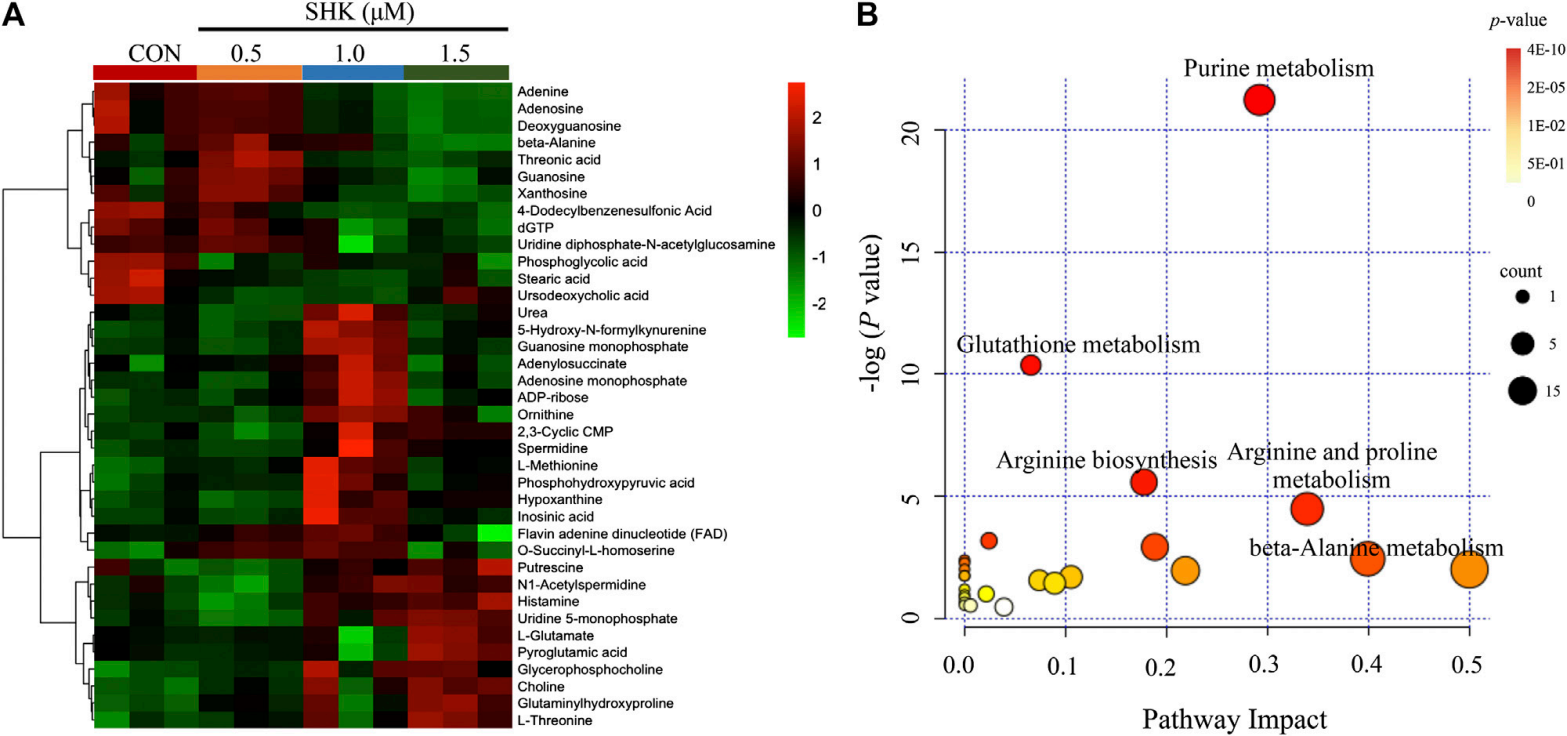
Metabolomic analysis of SW620 colon cancer cells. **(A)** Heatmap analysis of the differential metabolites between control group and shikonin-treated groups. **(B)** Summary of pathway analysis.

The metabolic profiles of SW620 cells were acquired under both positive and negative ionization modes by UPLC-MS/MS. A total of 6,524 ions in positive mode and 4,856 ions in negative mode were obtained. To obtain the difference of metabolic components among variable dose-treated groups, a multivariate statistical analysis method was used to screen the metabolites in 48 h. The score plot from PCA and OPLS-DA in were significantly separated (Supplementary Figure S1), indicating that shikonin treatment induced obvious disturbance of intercellular metabolites, and the effect of shikonin was related to concentration. In addition, significant differential variables contributing to the separation were screened based on the VIP > 1 and FDR < 0.05, and different metabolites were subsequently identified through matching MS/MS fragments with an online database. Finally, a total of 38 significant differentially expressed metabolites were identified, as shown in Table 1. These metabolites might account for the antitumor activity of shikonin on SW620 cells. A heatmap analysis was also performed to visualize the differences among 0.5, 1.0, and 1.5 μM shikonin-treated groups and control group (Figure 4A). Based on the distribution of colors, the metabolism of shikonin-treated groups showed significant changes compared with the control group, especially the group under high-concentration treatment (1.0 and 1.5 μM). The pathways disturbed by the above metabolites were mapped by MetaboAnalyst 4.0 (Figure 4B), suggesting that shikonin would affect SW620 cell viability through the following pathways: purine metabolism, glutathione metabolism, arginine biosynthesis, arginine biosynthesis, and beta-alanine metabolism. In particular, 13 differential metabolites involved in the purine metabolism were significantly regulated under the treatment of shikonin, which accounted for the highest impact among the perturbed pathways. Purine nucleotides play a vital role in the synthesis of DNA, RNA, and other metabolites during cell proliferation. The result suggested that the antitumor effect exerted by shikonin had a relationship with the decrease in the synthesis of purine nucleotides, further inhibiting the proliferation of cancer cells.

**TABLE 1:** Identification results of significant differential metabolites in cell samples.

Metabolite	MZ	RT	VIP	*p*-value^a^	FDR^b^	Mode
Guanosine monophosphate^c^	364.06442	1.22	1.78	3.28E-05	4.39E-03	Positive
Threonic acid^d^	135.03034	3.17	1.29	3.12E-04	9.55E-03	Negative
Adenine^d^	134.04642	2.73	6.86	6.18E-04	6.30E-03	Negative
Histamine^c^	112.08709	22.81	1.04	6.47E-04	6.20E-03	Positive
Xanthosine^d^	283.06979	4.08	1.58	1.68E-03	2.47E-02	Negative
N1-Acetylspermidine^c^	188.17543	0.86	2.23	2.73E-03	2.89E-02	Positive
Inosinic acid^d^	347.04152	1.23	4.80	2.86E-03	3.21E-02	Negative
Adenosine monophosphate^c^	346.05737	1.18	9.01	3.39E-03	3.30E-02	Negative
Deoxyguanosine^c^	266.09071	2.73	4.00	3.40E-03	3.32E-02	Negative
Ornithine^d^	131.08181	0.90	1.42	4.07E-03	3.40E-02	Negative
Uridine 5′-monophosphate^c^	347.02444	1.08	1.09	4.46E-03	3.25E-02	Positive
5-Hydroxy-N-formylkynurenine^c^	251.06913	1.44	1.05	4.87E-03	3.25E-02	Positive
Pyroglutamic acid^d^	130.04983	0.91	2.66	4.87E-03	3.25E-02	Positive
Adenosine^d^	312.09640	2.73	11.02	5.63E-03	4.80E-02	Negative
Guanosine^d^	282.08565	3.20	6.69	9.97E-03	4.36E-02	Negative
4-Dodecylbenzenesulfonic acid^c^	325.18587	23.19	2.01	1.08E-02	4.36E-02	Negative
L-Methionine^d^	150.05809	1.43	1.79	1.09E-02	4.23E-02	Positive
L-Glutamate^d^	148.06014	0.91	4.82	1.25E-02	4.40E-02	Positive
Adenosine diphosphate ribose^c^	558.06761	0.98	1.69	1.28E-02	4.36E-02	Negative
Ursodeoxycholic acid^c^	391.28344	21.98	3.95	1.51E-02	4.59E-02	Positive
Phosphoglycolic acid^c^	156.99040	15.55	2.32	1.67E-02	4.62E-02	Positive
β-Alanine^c^	134.04642	1.18	4.37	1.97E-02	4.41E-02	Negative
Urea^d^	159.02749	1.56	2.27	2.03E-02	4.82E-02	Positive
Stearic acid^d^	283.26555	21.64	1.14	2.08E-02	4.47E-02	Negative
Choline^d^	104.10728	0.89	5.83	2.21E-02	4.90E-02	Positive
Glutaminylhydroxyproline^c^	258.10961	0.90	4.61	2.26E-02	4.87E-02	Positive
O-Succinyl-L-homoserine^c^	218.06749	2.35	1.03	2.32E-02	4.47E-02	Negative
2′,3′-Cyclic cytidine monophosphate^c^	304.03553	1.00	1.41	2.40E-02	4.51E-02	Negative
Putrescine^c^	89.50707	23.35	2.56	2.41E-02	4.94E-02	Positive
Hypoxanthine^d^	137.04560	1.56	3.39	2.73E-02	4.98E-02	Positive
L-Threonine^d^	120.06557	0.91	1.13	2.82E-02	4.98E-02	Positive
Spermidine^d^	146.16493	0.73	5.49	3.57E-02	4.98E-02	Positive
Flavin adenine dinucleotide (FAD)^c^	784.15553	5.59	1.40	3.61E-02	4.53E-02	Negative
Uridine diphosphate-N-acetylglucosamine^c^	606.07806	0.99	9.86	3.62E-02	4.66E-02	Negative
Phosphohydroxypyruvic acid^c^	185.16457	1.92	1.36	4.08E-02	4.98E-02	Positive
dGuanosine triphosphate (dGTP)^c^	505.99113	2.01	4.26	4.20E-02	4.66E-02	Negative
Adenylosuccinate^c^	462.06931	3.86	5.84	4.32E-02	4.86E-02	Negative
Glycerophosphocholine^c^	258.10964	1.45	1.80	4.83E-02	4.98E-02	Positive

a *p*-values are calculated from a one-way ANOVA.

^b^FDR value was obtained from the adjusted *p*-value using Benjamini–Hochberg method.

^c^Represents metabolites that were identified by the MS/MS spectrum.

^d^Represents metabolites that were identified by commercial standards.

### Transcriptomics Analysis Results of Shikonin-Treated SW620 Colon Cancer Cell

**FIGURE 5:**
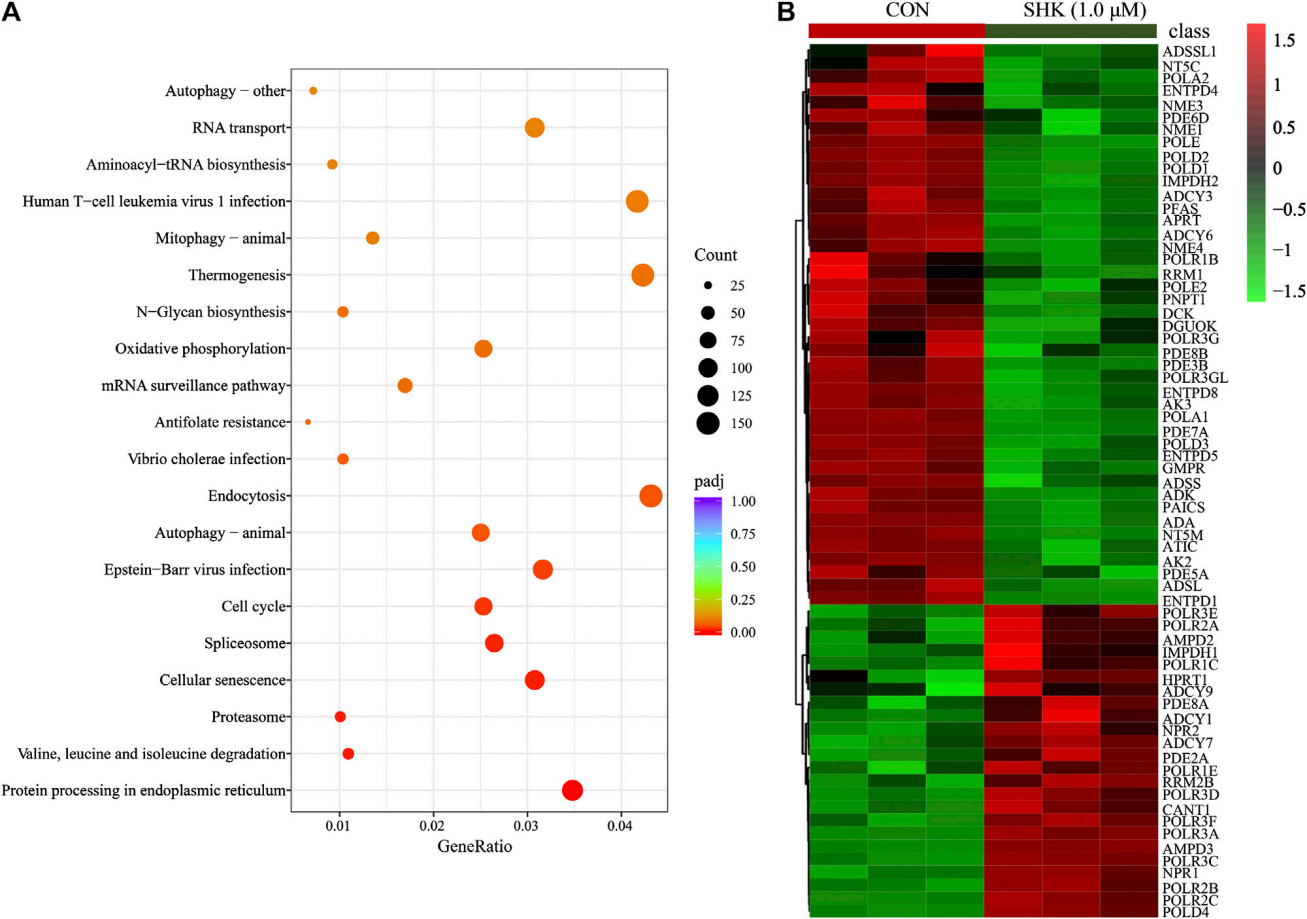
KEGG pathway analysis of transcriptomics data **(A)** and the heatmap of 85 differentially expressed genes involved in purine metabolism **(B)**.

Compared with the control group, a total of 10,409 differentially expressed genes were detected. To better understand the molecular function affected by shikonin, gene annotation enrichment analysis was then performed by GO database, which is an extensively used database for annotating genes. After mapping the differentially expressed genes to three terms in the GO database, the genes regulated by shikonin in SW620 cells could be mapped to biological processes (BP) for proteasomal protein catabolic process, ubiquitin-dependent protein catabolic process, and cell cycle G2/M phase transition; cell components (CC) for nuclear speck, chromosome centromeric region, and spliceosomal complex; and molecular functions (MF) for cadherin binding, ribonucleoprotein complex binding, and ubiquitin protein transferase activity (Supplementary Figure S2). Meanwhile, KEGG was also used for understanding high-level functions and utilities of the biological system (http://www.genome.jp/kegg/). The top 30 relevant pathways with *p* < 0.05 were shown in Figure 5A. The transcriptomics analysis showed that genes related to cell cycle transition were significantly regulated, especially in the G2/M phase, which corresponds to the result concluded by the flow cytometer. In addition, 85 genes involved in the purine metabolism were significantly regulated in the shikonin-treated group, and the heatmap analysis was performed to visualize the differences between the shikonin-treated group and control group in 24 h (Figure 5B).

### Integrated Metabolomics and Transcriptomics Data

**FIGURE 6:**
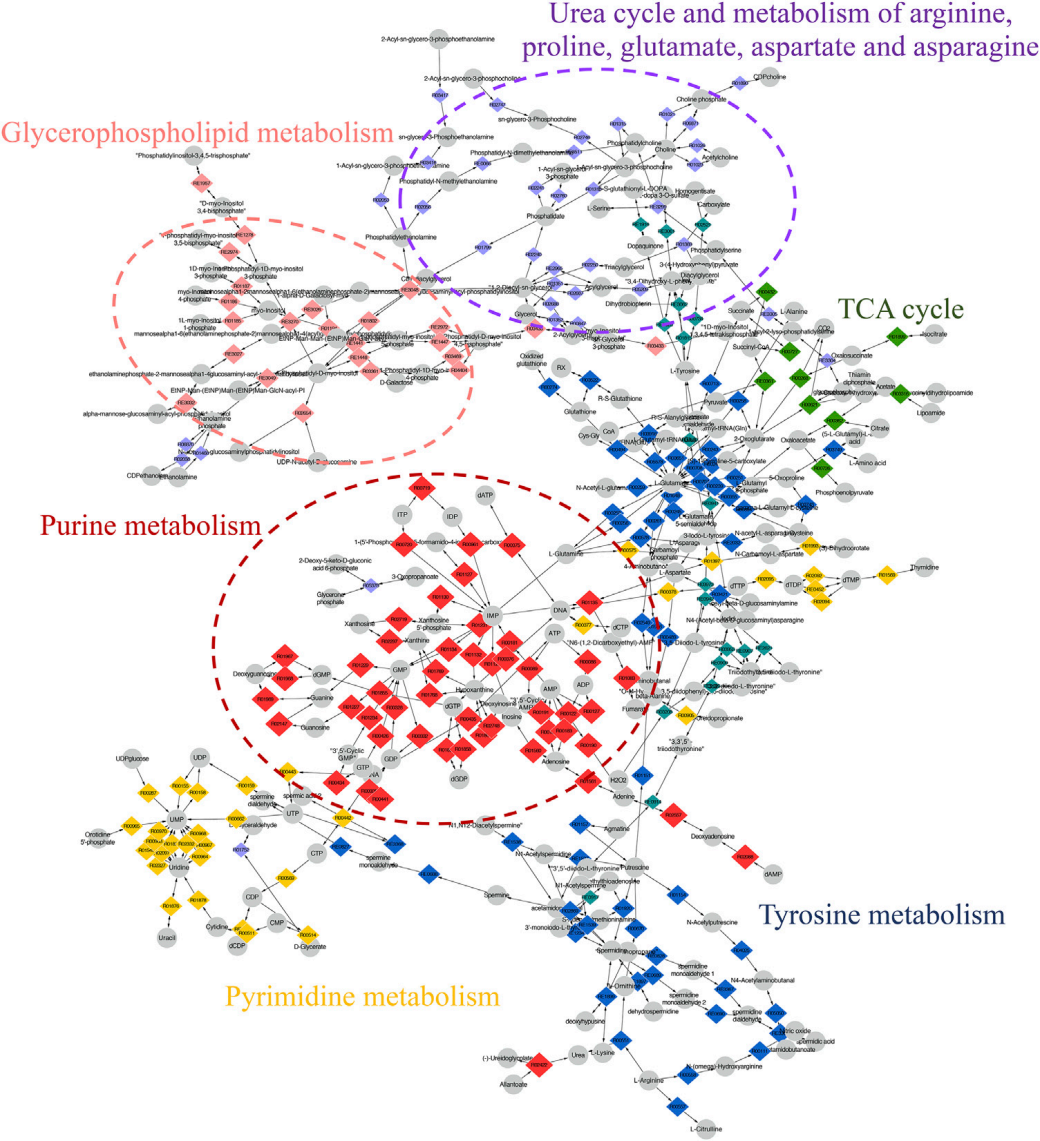
Scheme of perturbed metabolic pathways from metabolomics and transcriptomics integration. Compound reaction networks of the metabolites and genes were visualized using MetScape. Metabolites (circles) and reaction (diamond) are mainly enriched to six pathways.

**FIGURE 7:**
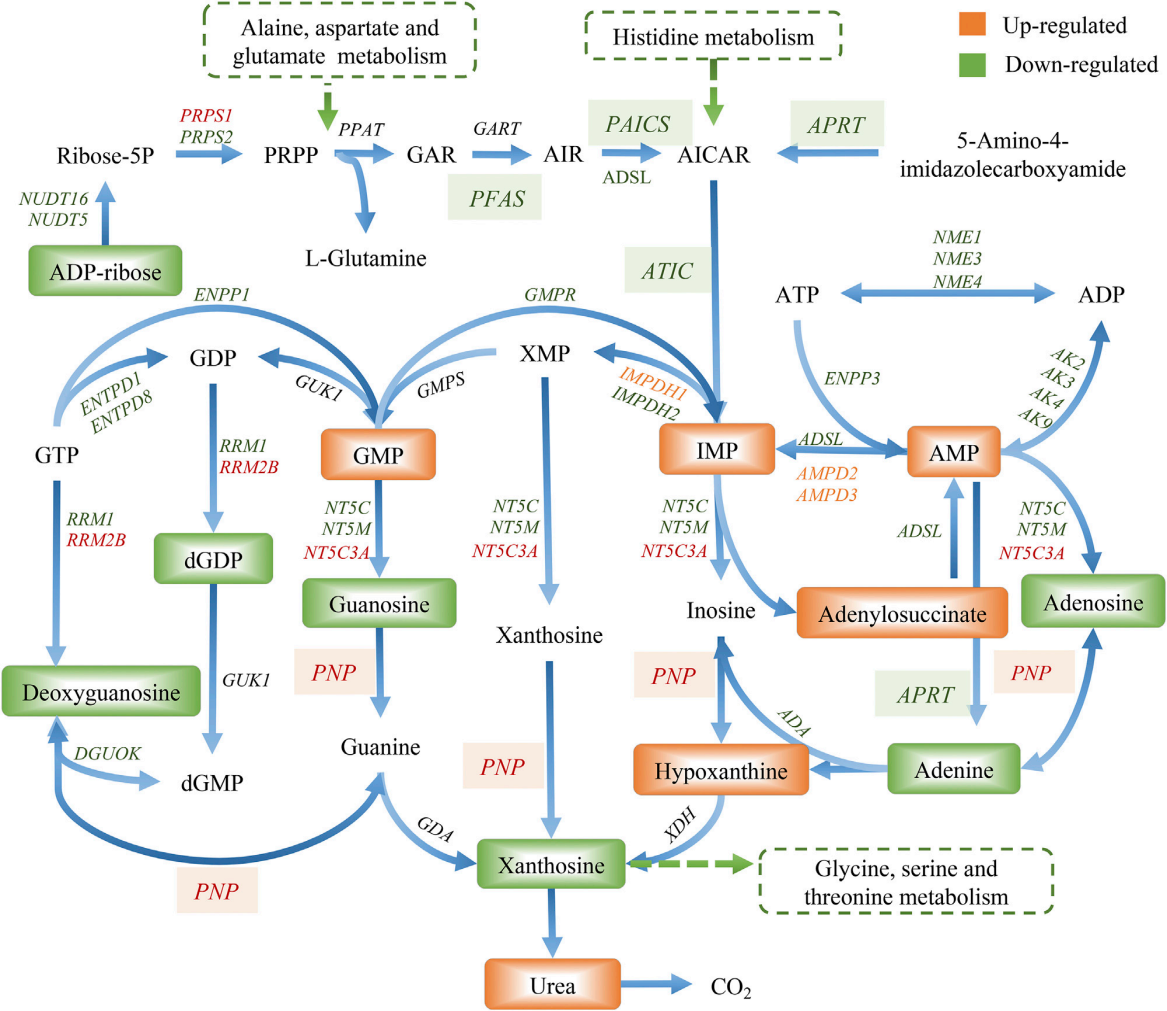
Graph of differentially expressed metabolites and genes involved in purine metabolism.

The compound reaction network was generated based on the transcriptomic and metabolomic data to interpret the potential relationship between the differentially expressed metabolites and genes. A global perturbed pathway network (SHK vs. CON) was formed with the differentiated metabolites and genes (Figure 6). The integrated analysis indicated that the disorder of the purine metabolism, glycerophospholipid metabolism, urea cycle, and metabolism of arginine, proline, glutamate, aspartate, and asparagine in SW620 colon cancer cells was involved in the antitumor effect of shikonin. In particular, the purine metabolism revealing an obvious differentiation in both metabolomic and integrated omics profiles between the shikonin-treated group and the control group was inferred as the major pathway involved in the shikonin therapeutic effect. A total of 13 metabolites and 85 genes were identified participating in the purine synthesis and metabolism. The scheme of regulated metabolites and related genes under the treatment is summarized in Figure 7. The results showed that the salvage and *de novo* purine synthesis was significantly inhibited after treating with shikonin, and the demand for adenosine triphosphate (ATP) was also down-regulated compared with the control group.

### Antitumor Activity of Shikonin and Biochemical Analysis *In Vivo*

**FIGURE 8:**
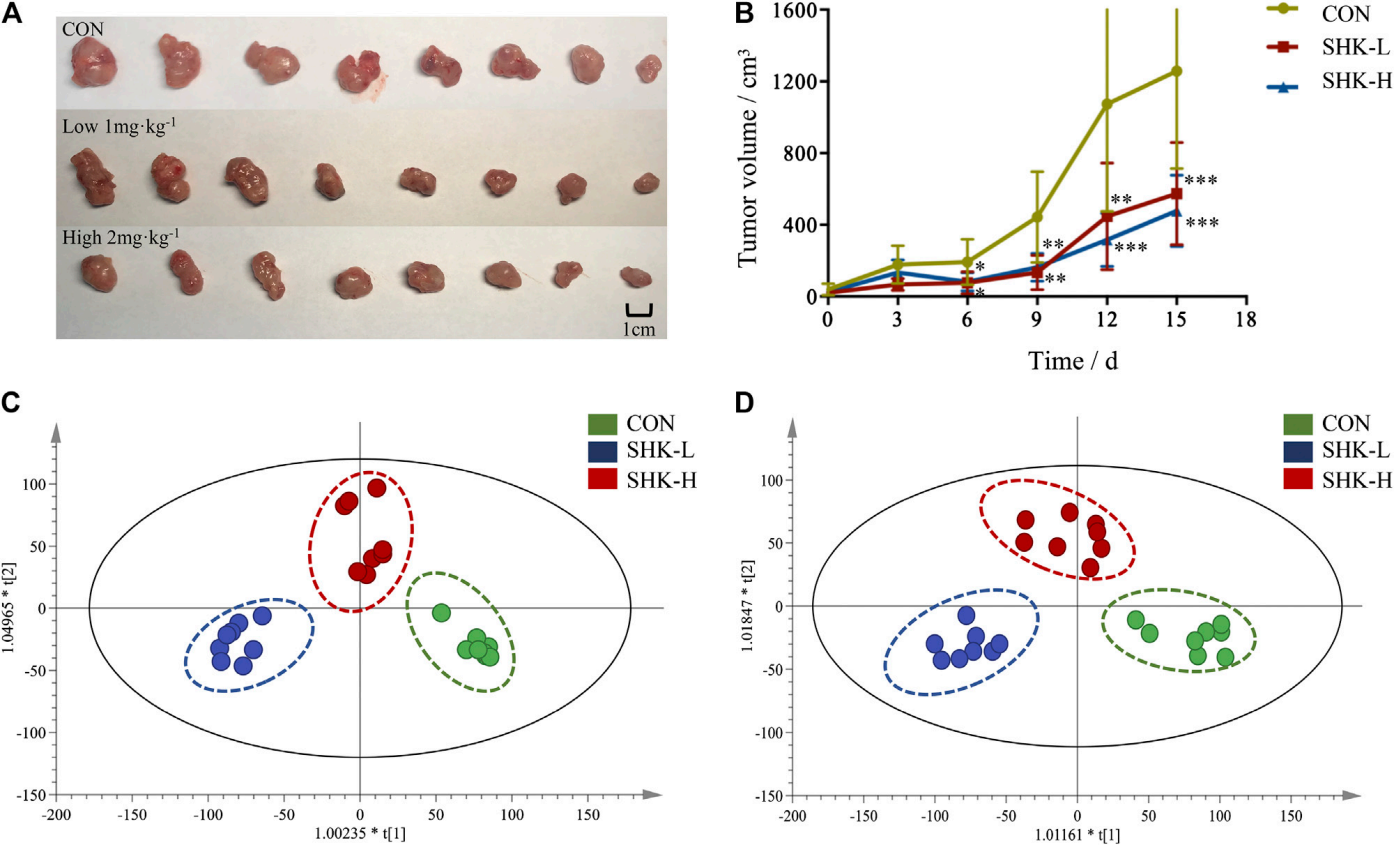
Antitumor activity of shikonin in the xenograft mouse model. **(A)** Photograph of the tumor volume in three groups. **(B)** Tumor inhibitory rate of shikonin in nude mice ($\bar{x}\pm s$, *n* = 8). *p < 0.05, **p < 0.01, ***p < 0.001 vs. control group only. **(C)** Score plot of OPLS-DA in positive mode from tumor tissue metabolomics data. **(D)** Score plot of OPLS-DA in negative mode from tumor tissue metabolomics data.

**FIGURE 9:**
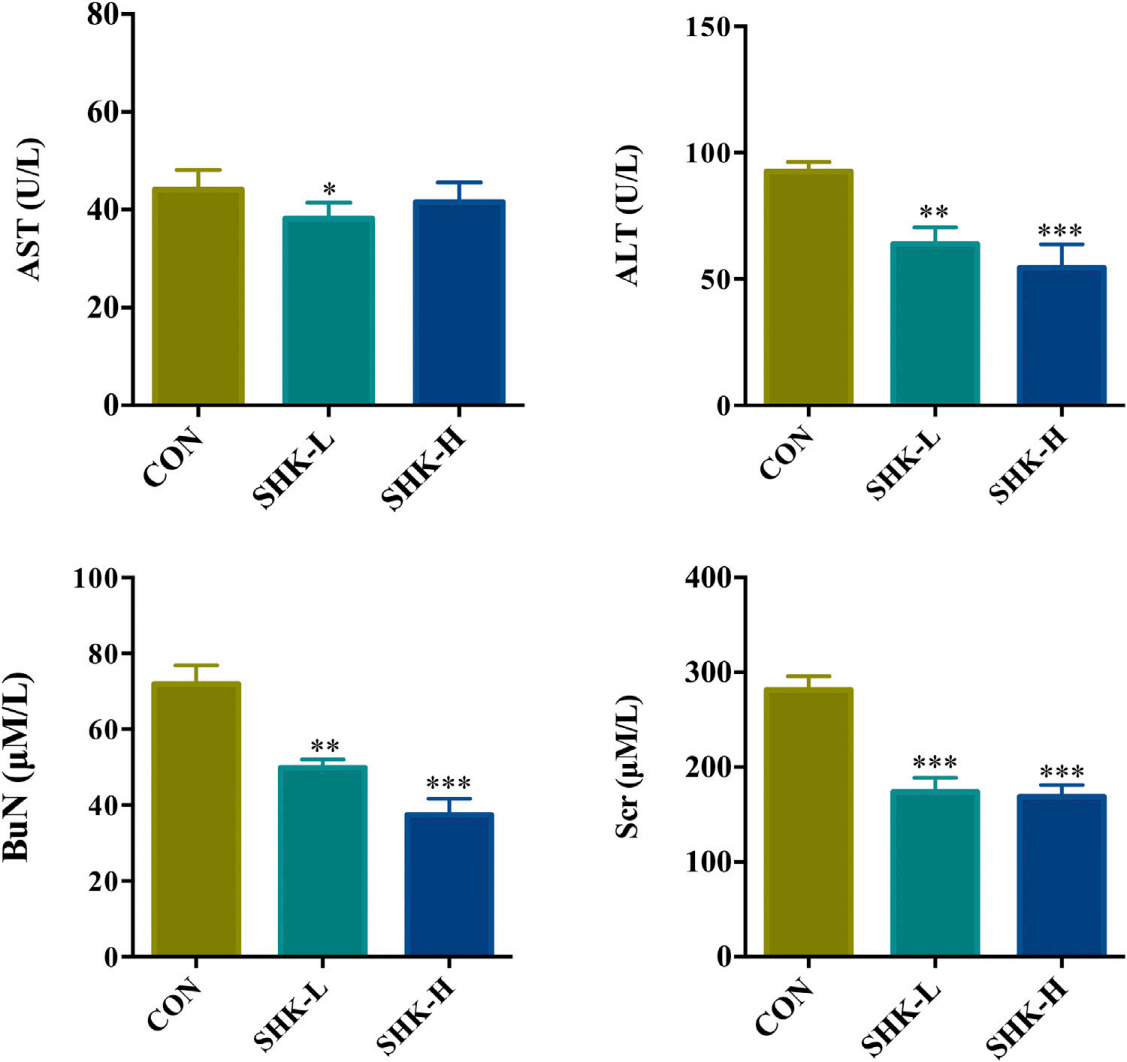
Effects of shikonin on liver enzymes (ALT and AST) and kidney functions (BuN and Scr) in nude mice with xenograft tumor. Each bar with vertical line represents mean ± SEM of eight mice per group. *p < 0.05, **p < 0.01, ***p < 0.001 vs. control group only.

To evaluate the potential effect of shikonin on colon cancer *in vivo*, a xenograft model in nude mice using the SW620 cell line was established. As shown in Figure 8A, both concentrations of the shikonin induced a significant reduction in tumor volume (*p* < 0.05). Meanwhile, the tumor masses were measured once every 3 days, and the results presented in Figure 8B demonstrated that shikonin could inhibit the tumor growth, and the tumor inhibitory rate of shikonin was 38.35 and 42.16% when administered at 1 or 2 mg/kg, respectively. As expected, mice under high-concentration treatment induced a highly significant reduction in tumor volume (*p* < 0.001) in a shorter time compared with the low-concentration group, which indicated the antitumor effect of shikonin in a dose-dependent manner. In addition, the shikonin-treated mice showed a decrease in serum AST, ALT, BUN, and Scr levels compared with those in the control group (Figure 9), which suggested that shikonin could alleviate the liver and kidney dysfunction in tumor xenograft mice.

### Metabolomic Analysis Results of Tumor Tissues

**FIGURE 10:**
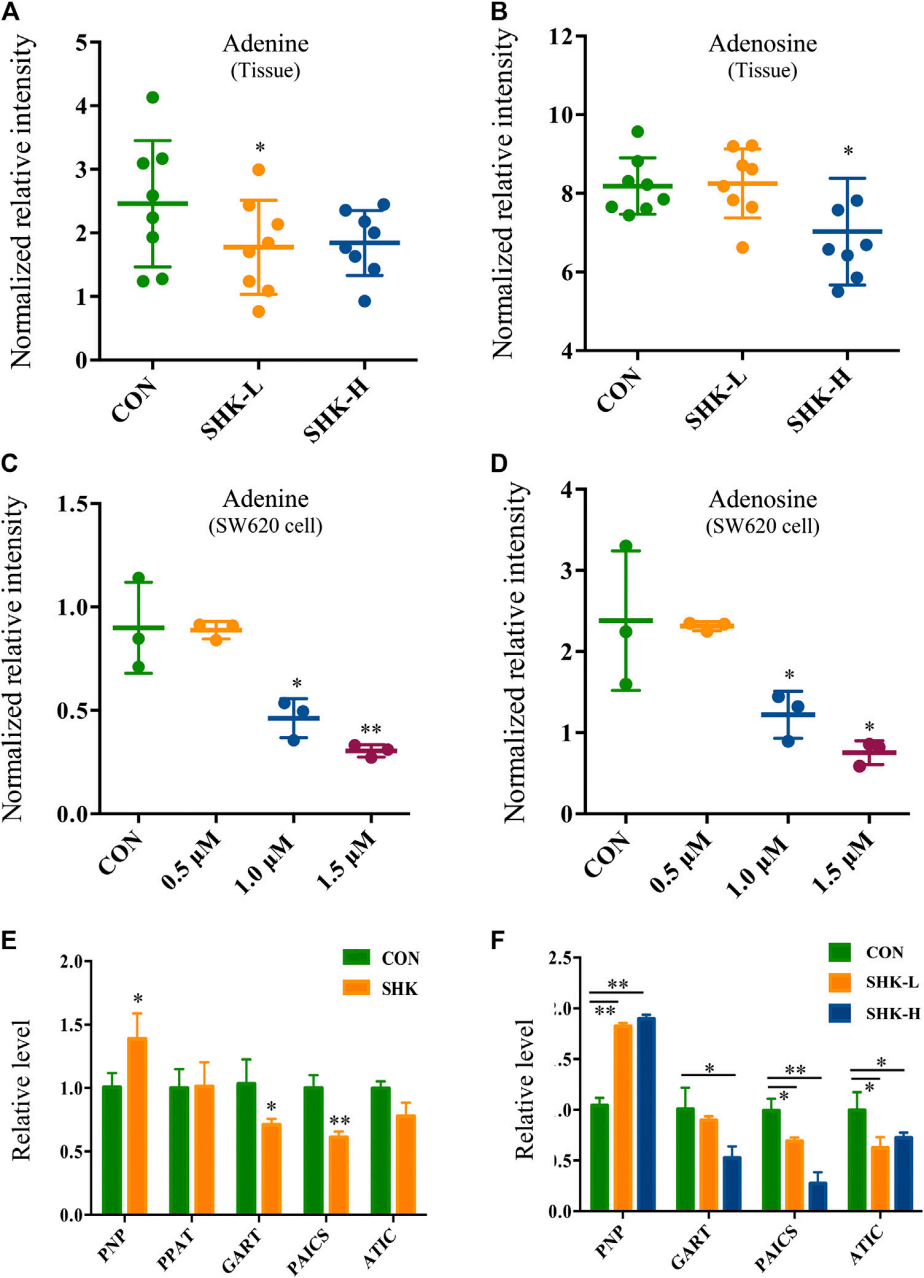
Expression level of adenine, adenosine, and purine-related genes in SW620 cells and tumor tissues. Relative intensity of adenine in tumor tissues **(A)** and SW620 cells **(C)**. Relative intensity of adenosine in tumor tissues **(B)** and SW620 cells **(D)**. Relative level of purine-related genes in SW620 colon cancer cells **(E)** and tumor tissues **(F)**.

To further reveal the perturbation of shikonin therapeutic intervention on colon cancer *in vivo*, the metabolomic profiles in three groups were evaluated with OPLS-DA score plots in positive and negative modes, which presented the best separation of control and drug experimental groups. The cross-test parameters R^2^X, R^2^Y, and Q^2^ values of the OPLS-DA model were 0.949, 0.992, and 0.776 and 0.992, 0.911, and 0.617 in positive and negative modes, respectively (Figures 8C,D), which suggested the good fitness and prediction of the established model. Based on the criteria of VIP > 1 in the OPLS-DA model, FC > 1.2 or <0.8 and *p* < 0.05 in Student’s *t*-test, a total of 23 significant differential metabolites were identified. As shown in Supplementary Table S1, these metabolites might account for the antitumor activity of shikonin in xenograft tumors. The pathways disturbed by above metabolites suggested that shikonin would affect SW620 cell viability through the purine metabolism (*p* = 0.0027) and D-glutamine and D-glutamate metabolism (*p* = 0.047). In particular, adenine and adenosine joint in the purine metabolism differently expressed in both SW620 cells and tumor tissue under the treatment of shikonin. Both of them were down-regulated in comparison with the control group, which was consistent with the metabolomic results in cellular metabolomics (Figures 10A–D).

### Validation of mRNA Expression Changes

To further validate the differential expression of purine metabolism–related genes, qRT-PCR was performed to measure the expression level of genes in both SW620 cells and tumor tissues. PNP, PPAT, GART, PAICS, and ATIC were selected as representatives to be validated based on the integration analysis. In general, the expression of most genes analyzed by qPCR exhibited high consistency with the results from transcriptomics analysis. PNP (purine nucleoside phosphorylase), known as a key enzyme in the rescue purine synthesis pathway and catalyzing purine nucleoside phosphorylation between adenine and adenosine reversibly, was up-regulated in both shikonin-treated groups in SW620 cells and tumor tissues. PPAT, GART, PAICS, and ATIC in the *de novo* purine synthesis pathway decreased significantly in shikonin-treated groups (Figures 10E,F). In general, the expression of genes analyzed by qPCR exhibited high consistency with the results from transcriptomics analysis.

## Discussion

CRC is a great menace to the elderly generation, who are diagnosed with late-stage disease suffering from poor prognosis. Hence, chemotherapies have been continuously improved to increase survival rates (Wu, 2018). Shikonin, a naphthoquinone compound, is extensively reported to exert antitumor activity against various types of cancer *in vitro* and *in vivo* (Boulos et al., 2019). Although a diversity of antitumor mechanisms involved in shikonin has been explored, the studies were mostly limited to the specific molecular targets in traditional pathways, making the pharmacological mechanisms hard to interpret thoroughly. In the present study, shikonin was confirmed to suppress the proliferation of four colon cancer cell lines in a dose-dependent and a time-dependent manner (Figure 1). To further clarify the antitumor mechanisms prior to clinical use, a multi-omics approach, which provides a systematic profile of endogenous metabolic changes in response to drug treatment, was performed to provide insights in regard to the global perturbation of shikonin treatment. Metabolic pathways, including the purine metabolism, glutathione metabolism, and amino acid metabolism, were found to be influenced under the treatment with shikonin. In particular, the purine metabolism had the most significant impact both in metabolomics analysis and integrated analysis, and a total of 13 significantly changed metabolites and 85 differentially expressed genes were identified participating in the purine synthesis and metabolism (Figure 6), indicating that the purine metabolism may account for the major perturbed pathway resulting from shikonin.

Purine nucleotides, which participate in various biological processes, are constantly synthesized *de novo* in all cells (Lane and Fan, 2015). Increased supplement of nucleotide synthesis is necessary for DNA replication, RNA procession, and ribosome biogenesis during cell proliferation (Zhu and Thompson, 2019). Serving as a promising target, enzymes involved in the nucleotide metabolism have been chosen as an approach to selectively impair proliferating cells in cancer therapy, and the antimetabolites of the nucleotide metabolism have been widely used in clinics, such as 5-fluoro-2-deoxyuridine and methotrexate, the inhibitor to the thymidylate synthase and the dihydrofolate reductase, respectively (Hatse et al., 1999). Compared to control groups, shikonin-treated groups tended to have less energetic metabolites and damage in DNA as the down-regulation tendency in adenosine monophosphate (AMP), adenosine, and adenine, which derived from the energy metabolism, nucleic acids, and mitochondrial DNA (Figure 7). More than that, GART, PAICS, and ATIC, which participated in the *de novo* synthesis of purine, decreased significantly in shikonin-treated groups according to the result of transcriptomics analysis and PCR validation (Figure 10). Glycinamide ribonucleotide transformylase (GART) is a significant trifunctional enzyme participating in purine and pyrimidine synthesis (Nagamani and Erez, 2016). The inhibitor of GART has been reported exerting a cytotoxic and a cytostatic effect on HCT116, MCF7, and A549 cancer cells (Bronder and Moran, 2003). Hence, serving as a core enzyme in the nucleotide metabolism, GART showed a potentiality as a promising target to antiproliferative and antitumor drugs. Moreover, phosphoribosyl pyrophosphate amidotransferase (PPAT) and phosphoribosylaminoimidazole carboxylase and phosphoribosylaminoimidazolesuccinocarboxamide synthase (PAICS), two key enzymes participating in hypoxanthine nucleotide (IMP) biosynthesis via the *de novo* pathway were found to be decreased after shikonin treatment. The essential role of PAICS in prostate cancer (Meng et al., 2018) and bladder cancer (Chakravarthi et al., 2018) was previously demonstrated. As we all known, the aberrant cell cycle with uncontrolled cell proliferation is consider to be a hallmark of cancers (Hanahan and Weinberg, 2011). In the current study, the cell cycle arrest in the G2/M phase and the abnormal cell cycle transition in cellular transcriptomics were found in shikonin-treated group (Figure 2), potentially resulting from the decreased expression of PAICS, which contributed directly to purine supplement for efficient DNA replication by producing N-succinocarboxyamide-5-aminoimidazole ribonucleotide (SAICAR) (Goswami et al., 2015). Besides, disruption of the microtubule arrangements in SW620 cells indicates that shikonin may have an impact on the depolymerizing microtubule through directly interacting with tubulin.

Adenosine, a purine nucleoside which could be secreted more under metabolic stress or hypoxia during tumor pathogenesis (Muller-Haegele et al., 2014), was down-regulated both in colon cancer cells and tumor tissues under shikonin treatment (Figure 10). Adenosine is known to promote angiogenesis (Guerrero, 2018), and evidence suggests that excessive adenosine released into the extracellular environment exerts effects on cancer progression through its immunosuppressive functions (Vigano et al., 2019). Both angiogenesis promotion and antitumor immune response suppression in the tumor microenvironment could contribute to tumor growth and metastasis. In addition, various types of adenosine receptors are up-regulated in different human tumor cell lines, and tumor tissues and the expression levels of adenosine receptors are especially high in human metastatic tissues (Sek et al., 2018). Endogenous adenosine could stimulate tumor growth in colon cancer cells through adenosine receptor activation (Gessi et al., 2007). Adenosine, along with adenine, the raw material for adenosine synthesis, decreased in the shikonin-treated group, indicating the potential function may be related to the reduction of immune suppression in tumor.

**FIGURE 11:**
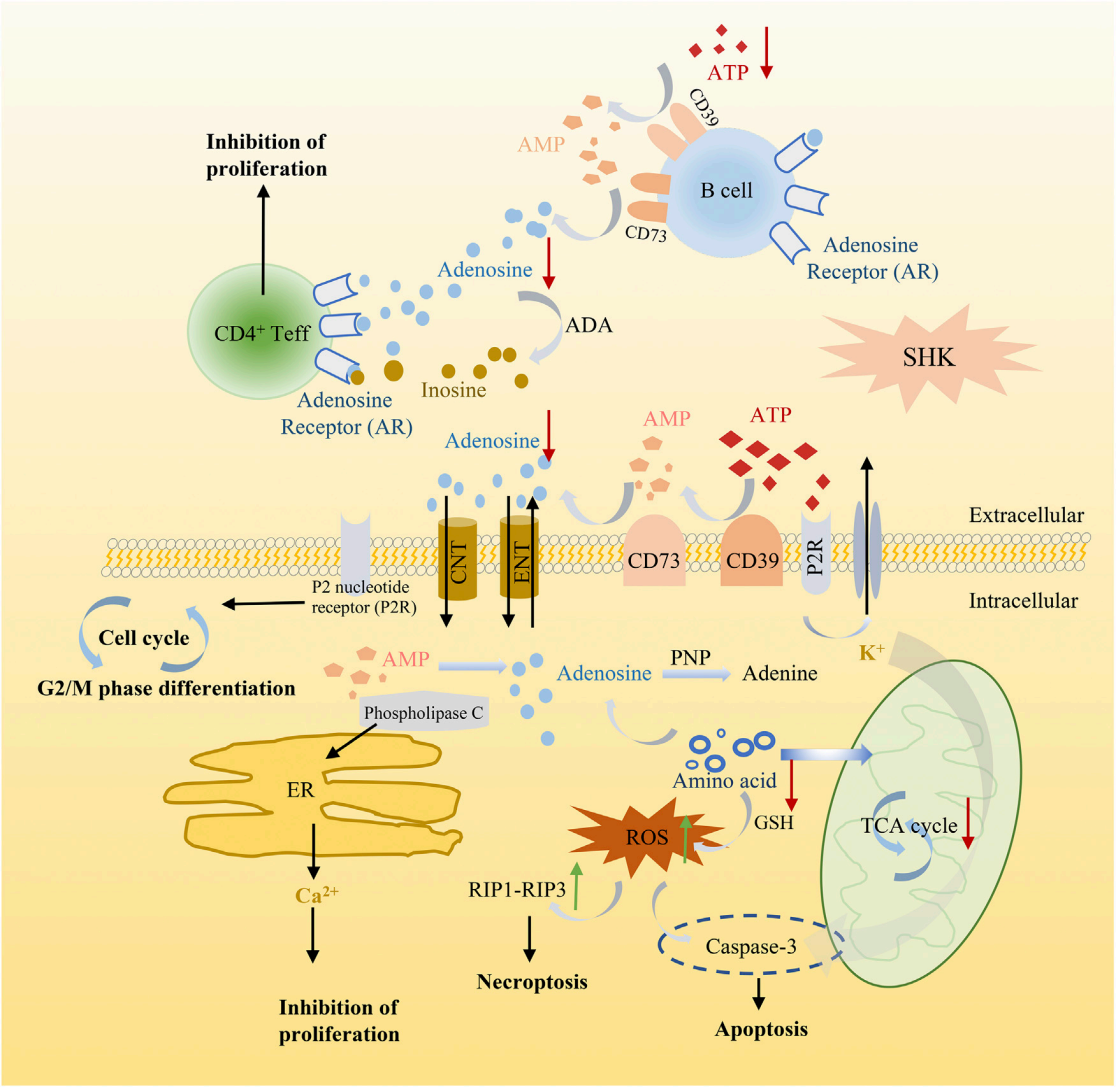
Schematic diagram illustrating the potential mechanism of shikonin in cancer cell function.

Meanwhile, in order to produce sufficient purine nucleotides to meet the commands for cell metabolism, a continuous supplement of carbon and nitrogen, which were derived from amino acids and metabolic intermediates, was needed (Newman and Maddocks, 2017). The alteration of several amino acids in shikonin treatment groups was observed as well, such as methionine, alanine, threonine, serine, and glutamate. These amino acids played a vital role in supplying precursor materials (Maddocks et al., 2017) for energy metabolism and biotransformation process. Nevertheless, the tricarboxylic acid cycle (TCA) was suppressed and glutathione metabolism was also disturbed after shikonin treatment, which indicated that oxidative stress may have an impact on shikonin-induced antitumor effect. As we all know, reactive oxygen species (ROS) is considered as a driving force for necroptosis and also participating in apoptosis (Zhang Z. et al., 2017). The kinase activity of receptor interacting protein 1 (RIP1), a key component in the necroptotic pathway, could be activated by ROS and further recruit RIP3 to form a functional necrosome, resulting in necroptosis (Zhang Y. et al., 2017). Based on these cumulative evidence, a schematic diagram of the potential mechanism exerted by shikonin was illustrated in cell function (Figure 11). Altogether, alteration above provides an expected systemic view of immune activation, energy deficiency, and oxidative damage that get involved in the therapeutic effect of shikonin.

## Conclusion

The present study demonstrated the cytotoxic effect mediated by shikonin against colon cancer both *in vitro* and *in vivo*. Integrated metabolomics and transcriptomics revealed that the antitumor activity may be related to the purine metabolism, amino acid metabolism, and glycerophospholipid metabolism. In addition, disturbance of the purine metabolism may account for the major mechanism resulting from shikonin antitumor activity. Altogether, this work provides new insights into exploring the potential mechanisms of the drug effect, which is important for pharmacological research and exploring novel chemotherapeutic agents in clinical cancer treatment.

## Data Availability Statement

The data sets generated for this study can be found in NCBI BioProject: PRJNA659884) (https://www.ncbi.nlm.nih.gov/bioproject/PRJNA659884).

## Ethics Statement

The animal study was reviewed and approved by Zhejiang Cancer Hospital Ethics Committee. Written informed consent was obtained from the owners for the participation of their animals in this study.

## Author Contributions

YC, ZC, and YW conceived and designed the study. YC provided resources and performed the majority of experiments. YG, XY, and JZ contributed experiments. All authors discussed the results. YC wrote the paper with inputs from all authors.

## Funding

This study was financially supported by the National Great New Drug Research and Development project (No. 2018ZX09201010) and the National Natural Science Foundation of China (No. 81302840).

## Conflict of Interest

The authors declare that the research was conducted in the absence of any commercial or financial relationships that could be constructed as a potential conflict of interest.
