# Supplementary material for: Integration of Transcriptomics and Metabolomics Reveals the Antitumor Mechanism Underlying Shikonin in Colon Cancer
Source: Front Pharmacol. 2020 Oct 22;11:544647. doi: 10.3389/fphar.2020.544647 (PMC7689381; doi:10.3389/fphar.2020.544647)
Supplement: Supplementary file 1 [file Image1_v1.pdf]

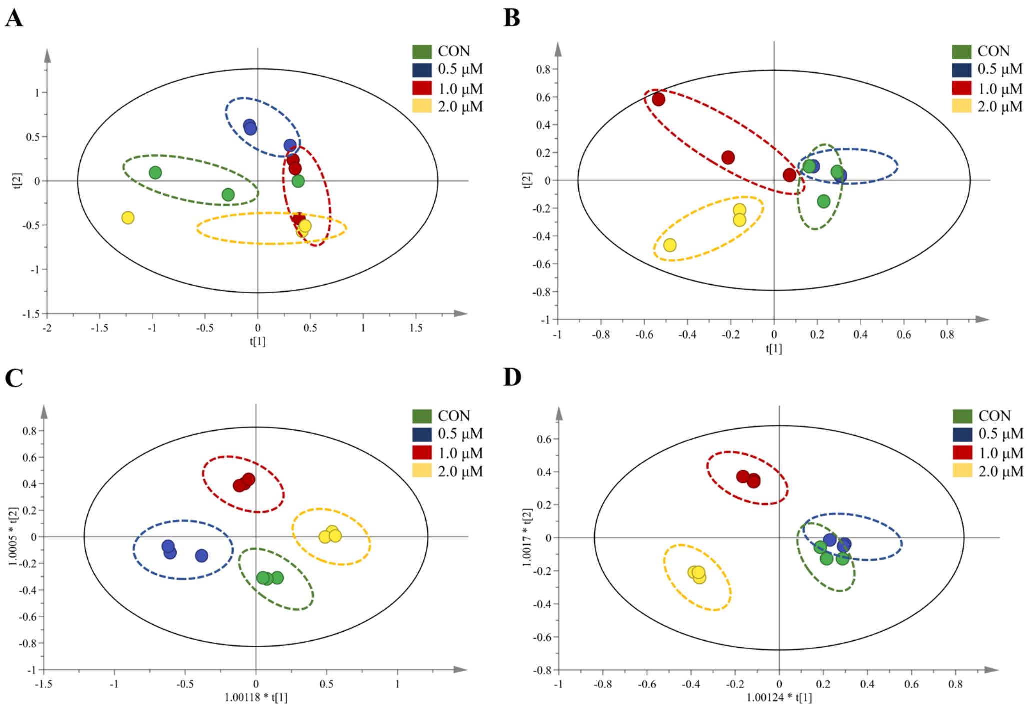

Figure S1. Scores plots of PCA and OPLS-DA from cellular metabolomics. (A) Scores plot of PCA in positive mode. (B) Scores plot of PCA in negative mode. (C) Scores plot of OPLS-DA in positive mode. (D) Scores plot of OPLS-DA in negative mode.
