# Supplementary material for: Integration of Transcriptomics and Metabolomics Reveals the Antitumor Mechanism Underlying Shikonin in Colon Cancer
Source: Front Pharmacol. 2020 Oct 22;11:544647. doi: 10.3389/fphar.2020.544647 (PMC7689381; doi:10.3389/fphar.2020.544647)
Supplement: Supplementary file 2 [file Image2_v1.pdf]

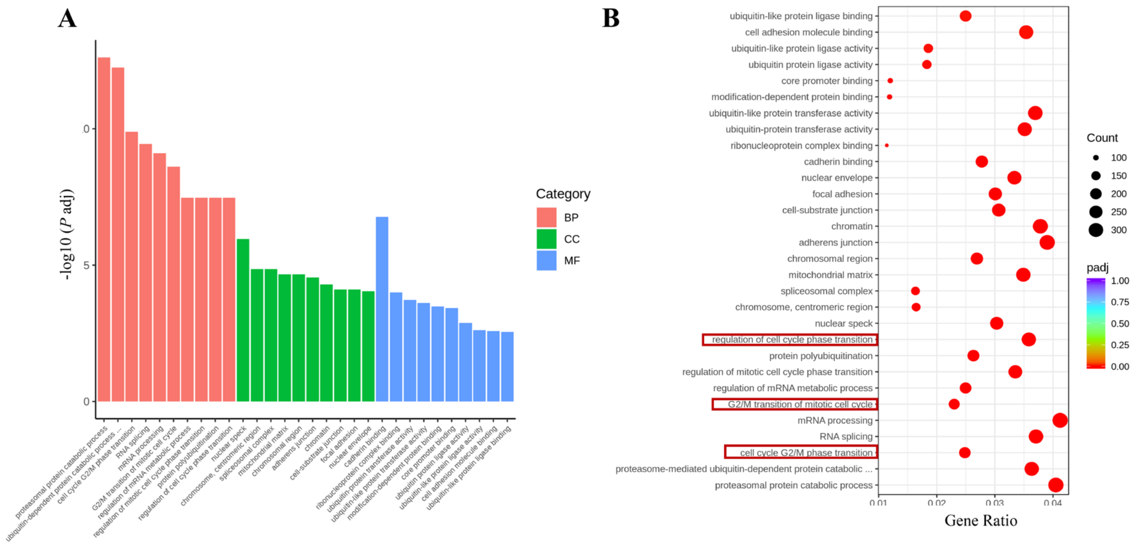

Figure S2. Transcriptomics analysis of Shikonin in SW620 cells. (A) GO enrichment analysis of differently expressed mRNAs in BP (Biological process), CC (Cellular Component) and MF (Molecular function). (B) The top 30 relevant terms in GO enrichment analysis from a comprehensive view.
