# Supplementary material for: Integration of Transcriptomics and Metabolomics Reveals the Antitumor Mechanism Underlying Shikonin in Colon Cancer
Source: Front Pharmacol. 2020 Oct 22;11:544647. doi: 10.3389/fphar.2020.544647 (PMC7689381; doi:10.3389/fphar.2020.544647)
Supplement: Supplementary file 3 [file Table1_v2.docx]

**Table S1.** Primers sequence for qPCR

| Genes | Forward | Reverse |
| --- | --- | --- |
| **PNP** | GCAGGGCAGGTTCCACAT | TCCGGTCGTAGGCATCAG |
| **PPAT** | GTGGGTGCTTCCTTCATC | TCACTCTGGGACTCGTGG |
| **GART** | CCATCTAAGGTGGACTGA | TGCTGGAATAATGCTGAC |
| **PAICS** | AAACAGAATGCTCCCAACT | AACCCAAGAACCAATGAA |
| **ATIC** | GTGGTGGAAGAGCCGAAG | GCAGGAGATAAGGCAAAC |
| **GAPDH** | GAAGGTGAAGGTCGGAGT | GAAGATGGTGATGGGA |
