# Supplementary material for: Integration of Transcriptomics and Metabolomics Reveals the Antitumor Mechanism Underlying Shikonin in Colon Cancer
Source: Front Pharmacol. 2020 Oct 22;11:544647. doi: 10.3389/fphar.2020.544647 (PMC7689381; doi:10.3389/fphar.2020.544647)
Supplement: Supplementary file 4 [file Table2_v2.docx]

**Table S1.** Identificaton of differential metabolites in tumor tissues.

|  |  |  |  |  | SHK-L vs CON | | | SHK-H vs CON | | |  |
| --- | --- | --- | --- | --- | --- | --- | --- | --- | --- | --- | --- |
| No. | Metabolites | M.Z. | R.T. | VIP | FC | p-value | FDR | FC | p-value | FDR | Mode |
| 1 | L-Ascorbic acid | 175.02416 | 70.61 | 13.77 | 1.65 | 2.27E+00 | 3.92E-02 | 0.00 | 6.86E+02 | 1.76E+03 | negative |
| 2 | Isodesmosine | 526.29044 | 559.66 | 7.39 | 0.44 | 1.91E-03 | 2.03E-02 | 0.18 | 1.82E-05 | 6.14E-04 | positive |
| 3 | Myristoylcarnitine | 372.30913 | 535.46 | 6.01 | 0.36 | 5.89E-03 | 3.10E-02 | 0.21 | 1.63E-03 | 5.27E-03 | positive |
| 4 | Acetylhomoserine | 162.07525 | 69.98 | 5.98 | 0.53 | 2.33E-03 | 2.16E-02 | 0.49 | 7.85E-04 | 3.15E-03 | positive |
| 5 | Allopurinol | 137.04522 | 102.56 | 5.93 | 0.63 | 1.26E-02 | 3.39E-02 | 1.55 | 5.93E-03 | 1.30E-02 | positive |
| 6 | Hexadec-2-enoyl carnitine | 398.32469 | 546.22 | 5.33 | 0.37 | 7.17E-03 | 3.10E-02 | 0.22 | 1.57E-03 | 5.10E-03 | positive |
| 7 | 3'-AMP | 346.05671 | 71.03 | 4.39 | 0.77 | 7.88E-01 | 1.65E-02 | 0.04 | 5.04E+02 | 4.09E+02 | negative |
| 8 | Adenosine | 268.10280 | 167.75 | 3.74 | 0.49 | 1.41E-02 | 3.42E-02 | 0.21 | 1.81E-04 | 1.37E-03 | positive |
| 9 | 3-methyl pyruvic acid | 103.03885 | 131.03 | 3.64 | 0.48 | 1.85E-01 | 1.12E-02 | 0.00 | 3.18E+02 | 2.31E+02 | negative |
| 10 | Deoxyguanosine | 312.09604 | 168.19 | 3.48 | 0.51 | 2.52E-01 | 5.66E-03 | 0.00 | 1.54E+02 | 1.67E+02 | negative |
| 11 | N-Oleoyl-L-Serine | 370.29346 | 511.19 | 2.80 | 0.51 | 5.46E-03 | 3.08E-02 | 0.54 | 7.47E-03 | 1.50E-02 | positive |
| 12 | Dodecanoylcarnitine | 344.27790 | 490.96 | 2.19 | 0.55 | 6.48E-03 | 3.00E-02 | 0.62 | 2.32E-02 | 3.24E-02 | positive |
| 13 | Glutaconic acid | 129.01828 | 63.10 | 1.94 | 0.80 | 6.27E-01 | 3.14E-02 | 0.00 | 8.03E+01 | 6.25E+01 | negative |
| 14 | D-Glutamic acid | 130.04943 | 70.78 | 1.90 | 0.63 | 5.79E-03 | 3.09E-02 | 0.58 | 2.16E-03 | 6.42E-03 | positive |
| 15 | Adenine | 136.06119 | 167.76 | 1.75 | 0.59 | 5.36E-03 | 3.09E-02 | 0.43 | 3.27E-04 | 1.75E-03 | positive |
| 16 | Leucyl-Arginine | 286.18550 | 540.43 | 1.62 | 0.40 | 2.53E-01 | 2.39E-02 | 0.01 | 1.64E+01 | 1.94E+01 | negative |
| 17 | N-Undecylbenzenesulfonic acid | 311.16959 | 838.43 | 1.54 | 0.34 | 2.36E-01 | 7.82E-03 | 0.00 | 1.23E+01 | 2.84E+01 | negative |
| 18 | Formylphosphonate | 110.98410 | 54.93 | 1.44 | 0.69 | 3.10E-01 | 2.35E-02 | 0.00 | 1.95E+01 | 2.74E+01 | negative |
| 19 | ADP | 426.02388 | 60.62 | 1.43 | 1.55 | 1.83E+00 | 4.59E-02 | 0.02 | 1.21E+01 | 2.43E+01 | negative |
| 20 | 3-Hydroxy-2-naphthoate | 187.04155 | 177.02 | 1.39 | 1.67 | 1.58E+00 | 7.59E-04 | 0.00 | 1.77E+01 | 2.10E+01 | negative |
| 21 | 12-Hydroxydodecanoic acid | 215.16524 | 547.93 | 1.30 | 0.50 | 5.23E-01 | 8.86E-03 | 0.02 | 3.95E+01 | 1.87E+01 | negative |
| 22 | Oleic acid | 281.24934 | 768.01 | 1.24 | 0.42 | 1.61E-01 | 5.42E-03 | 0.00 | 1.26E+01 | 1.12E+01 | negative |
| 23 | 3-hexenedioic acid | 145.04886 | 70.51 | 1.06 | 0.65 | 3.14E-03 | 2.53E-02 | 0.55 | 3.65E-04 | 1.89E-03 | positive |
